# Acid Suppression Use Among Infants in One Tertiary Children's Hospital in China, 2015–2018: A Retrospective Observational Study

**DOI:** 10.3389/fped.2021.679203

**Published:** 2021-05-21

**Authors:** Yue Zhou, Lina Xu, Haishaerjiang Wushouer, Aichen Yu, Ziyue Xu, Chaoyang Chen, Yimin Cui, Qinghong Lu, Xiaodong Guan, Luwen Shi

**Affiliations:** ^1^Department of Pharmacy Administration and Clinical Pharmacy, School of Pharmaceutical Sciences, Peking University, Beijing, China; ^2^Department of Pharmacy, Jiangxi Provincial Children's Hospital, Nanchang, China; ^3^International Research Center for Medicinal Administration (IRCMA), Peking University, Beijing, China; ^4^Department of Pharmacy, Peking University First Hospital, Beijing, China; ^5^Institute of Clinical Pharmacology, Peking University, Beijing, China

**Keywords:** acid suppressions, histamine-2 receptor antagonists, proton pump inhibitors, infants, off-label drug use

## Abstract

Clinical guidelines emphasized that physicians should be cautious when prescribing acid suppressions to infants. Histamine-2 receptor antagonists (H2RAs) and proton pump inhibitors (PPIs) are not approved for use in infants aged below 2 years in China. We investigated H2RA/PPI use in infants aged below 2 years hospitalized between 1st January 2015 and 31st December 2018 in a Chinese tertiary children's hospital. Our study observed that H2RAs/PPIs were frequently prescribed with a prevalence of 4.4% (7,158/162,192). The frequency of PPI use was over two-fold than that of H2RA use (71.9%, 5,148/7,158; 28.1%, 2,011/7,158). H2RAs/PPIs were commonly used to treat infants without digestive system diseases (57.5%, 4,118/7,158). Further studies are urgently needed to evaluate the effectiveness and safety of H2RAs/PPIs in infants.

## Introduction

Histamine-2 receptor antagonists (H2RAs) and proton pump inhibitors (PPIs) are acid suppressions for gastric acid-related disorders. However, considering the unique characteristics of infant stomach and the different reflux-type symptoms, guidelines emphasized that physicians should be cautious when prescribing H2RAs/PPIs to infants (1–6). The effectiveness of H2RAs/PPIs to treat gastric acid-related disorders in infants was still questioned (7, 8). Studies indicated a potential association between H2RAs/PPIs and an increased risk of respiratory tract or gastrointestinal infections, *Clostridium difficile* infection, and hypomagnesemia in infants (8–11). Nonetheless, H2RAs/PPIs were prescribed to infants frequently and the use had increased dramatically for the past two decades (12–16). Up to now, H2RAs/PPIs are not approved for children in China. Though previous studies highlighted the overuse of PPI medication in adults and the multiple potentially serious side effects (17–19), few studies targeted H2RA/PPI off-label use in infants. Hence, we described H2RA/PPI off-label use trends in infants, and evaluate the association between patient characteristics and diagnoses with H2RA/PPI selection.

## The Trend of H2RA/PPI Use in China

**Figure 1:**
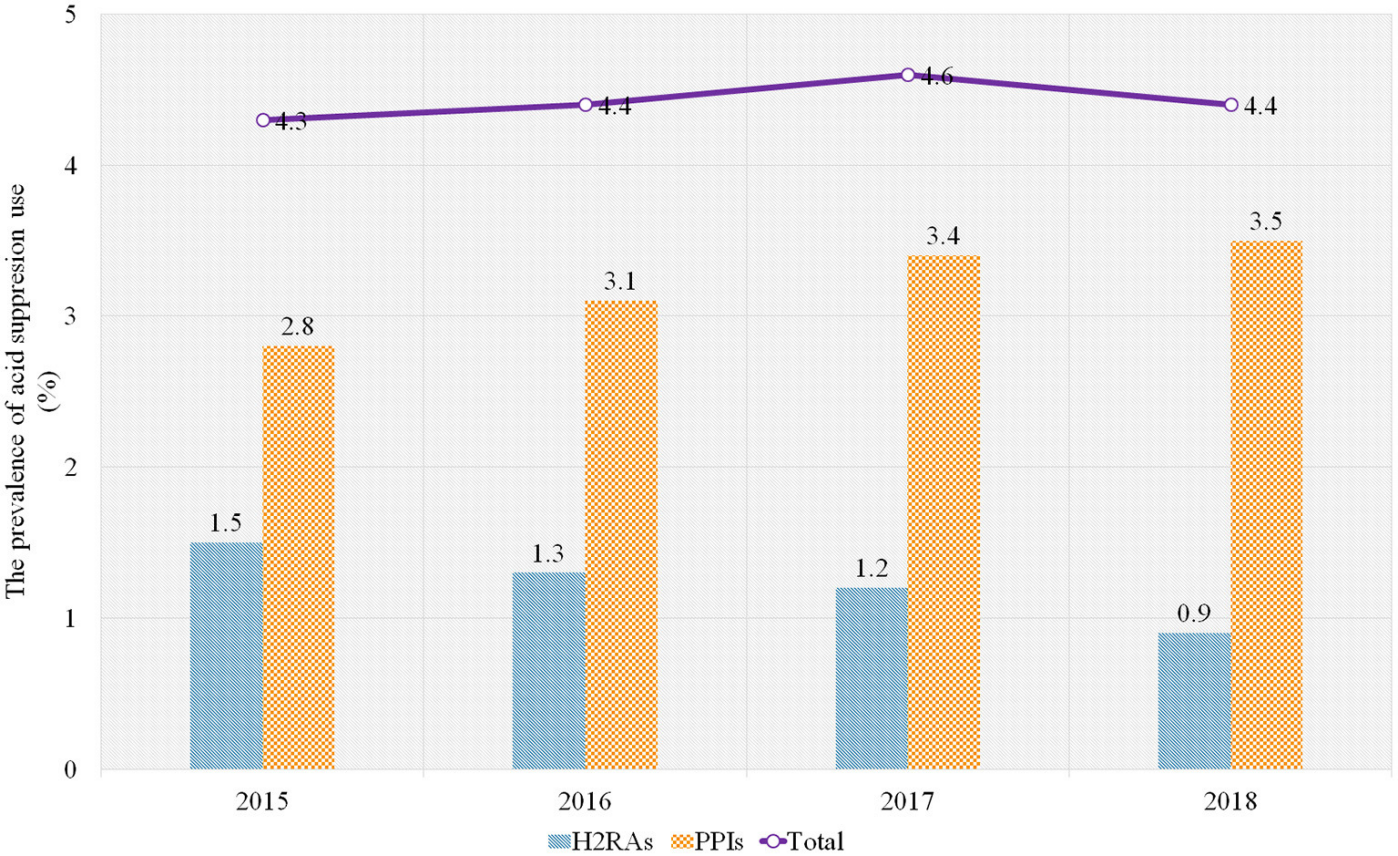
The prevalence of H2RA/PPI use in the sample hospital, 2015–2018. PPIs, proton pump inhibitors; H2RAs, Histamine-2 receptor antagonists.

We calculated the prevalence of H2RA/PPI use (measured by the percentage of admissions treated with H2RAs/PPIs of all admissions) and the frequency of H2RA/PPI use (measured by the number of admissions treated with H2RAs/PPIs) from inpatients aged below 2 years between 1st January 2015 and 31st December 2018 in a tertiary children's hospital in China. Overall, of 162,192 admissions of patients aged below 2 years in the databases during the study period, 7,158 (4.4%) admissions of the infants treated with H2RAs/PPIs were included. The frequency of PPI use was over 2-fold than that of H2RA use (71.9%, 5,148/7,158; 28.1%, 2,011/7,158). Omeprazole and Cimetidine were the most commonly prescribed PPI and H2RA, respectively (69.6%, 4,981/7,158; 28.1%, 2,010/7,158). During the 4-year study period, the prevalence of total H2RA/PPI use was 4.3%, 4.4%, 4.6%, and 4.4%, respectively. The prevalence of PPI use increased from 2.8% to 3.5% while H2RA use decreased from 1.5% to 0.9% (Figure 1, Supplementary Table 1).

## Factors Associated with H2RA/PPI Selection

The factors associated with H2RA/PPI selection included characteristics and diagnoses of the patients. Characteristic data was retrieved from the hospital medication records, including the admission number of the patients, time of admission, gender, age, medical insurance, diagnoses, medication information, and the number of admissions of patients aged below 2 years. The diagnoses in the hospital were coded using the International Statistical Classification of Diseases, 10th Revision (ICD-10) diagnostic codes (20). The logistic regression analyses were conducted and odds ratios (ORs) and estimates were presented with their 95% confidence intervals (CIs). The regression demonstrated infants aged above 28 days were more likely to be prescribed PPIs than H2RAs (OR = 21.6, 95% CI 17.9–26.1, *P* < 0.001; OR = 7.6, 95% CI 6.3–9.2, *P* < 0.001). Infants covered by the New Rural Cooperative Medical Insurance (NRCMS) or without medical insurance were more likely to use H2RAs than PPIs (OR = 0.7, 95% CI 0.6–0.9, *P* < 0.001; OR = 0.8, 95% CI 0.7–0.9, *P* = 0.001). Compared with H2RAs, there was a positive correlation between diagnoses including digestive system diseases and PPI use (OR = 2.0, 95% CI 1.8–2.3, *P* < 0.001) (Table 1).

**Table 1:** Characteristics and diagnoses of patients prescribed PPIs compared with those prescribed H2RAs.

**Variables**	**Inpatient**			
	***N*** **=** **7,158**, ***n*** **(%)**			
	**PPIs Cohort**	**H2RAs Cohort**	**PPIs vs. H2RAs OR (95% CI)**	***P-value***
**Gender**				
Male	3,339 (46.6)	1,282 (17.9)	1.0 (reference)	
Female	1,809 (25.3)	728 (10.2)	1 (0.9–1.1)	0.81
**Age**				
<28 days	230 (3.2)	798 (11.1)	1.0 (reference)	
(28days, 1 year)	3,568 (49.8)	612 (8.5)	21.6 (17.9–26.1)	<0.001
(1 year, 2 years)	1,350 (18.9)	600 (8.4)	7.6 (6.3–9.2)	<0.001
**Insurance**				
URBMI	1,675 (23.4)	403 (5.6)	1.0 (reference)	
NRCMS	1,620 (22.6)	470 (6.6)	0.7 (0.6–0.9)	<0.001
None	1,853 (25.9)	1,137 (15.9)	0.8 (0.7–0.9)	0.001
**Diagnoses**				
No digestive system diseases	2,807 (39.2)	1,311 (18.3)	1.0 (reference)	
With digestive system diseases	2,341 (32.7)	699 (9.8)	2 (1.8–2.3)	<0.001

*URBMI, the Urban Residents' Basic Medical Insurance; NRCMS, the New Rural Cooperative Medical Insurance; PPIs, proton pump inhibitors; H2RAs, Histamine-2 receptor antagonists; OR, odds ratio; CI, confidence interval*.

## Discussion

We observed that although H2RAs/PPIs were not approved in China for infants, they were frequently prescribed for the population, commonly being used to treat the infants without digestive system diseases. Compared to placebo, H2RAs/PPIs possess uncertain effectiveness in infants. Meanwhile, H2RAs/PPIs could induce infections through changing the stomach acidity that functioned as the barrier to detrimental pathogens through the gastrointestinal tract host defense system (11, 21). Evidences in adults also highlighted that long-term PPI use was associated with adverse events including bone fracture, renal disease, cardiac disease, etc. (22). Considering the uncertain effectiveness and potential safety problems of H2RAs/PPIs, more cautions should be taken when prescribing them for the younger population.

The trend of H2RA/PPI use in the sample hospital was relatively stable over time, and the prevalence of PPI use was higher than that of H2RA use. Studies found PPIs were superior to H2RAs in symptom control, such as higher rates of healing of erosive or histologic esophagitis (23–26). Guidelines recommended PPIs as a first-line treatment for server gastric acid-related diseases in infants while H2RAs were suggested when PPIs were unavailable or contra-indicated or the symptom was mild to moderate (5, 27). This may explain the observation of higher frequency of PPI use in our study. Although PPIs were considered to offer advantages over H2RAs in the pediatric population, we observed that H2RAs were still used in nearly one-third of the infants. The infants without medical insurance or covered by NRCMS were more likely to be prescribed with H2RAs. Healthcare expenditure of PPIs was higher than that of H2RAs in the pediatric population (28). Different from their counterparts covered by the Urban Residents' Basic Medical Insurance (a more beneficial schemes), infants covered by NRCMS or without medical insurances were more likely subjected to healthcare expenditure burden and tended to be treated with H2RAs (29).

The main indications of H2RAs/PPIs approved were to treat gastroesophageal reflux disease and to heal and prevent gastroduodenal ulcers. However, our study observed the proportion of admissions diagnosed without digestive system diseases, over one-half, was unexpected; the respiratory system diseases were the most common diagnoses. Respiratory system diseases, such as asthma and acute upper respiratory tract infection (URTI) are common disorders in children. Gastroesophageal reflux is prevalent among children suffering from asthma (5, 30–32). However, there were no significant differences in asthma symptom remission or pulmonary function improvement but a higher risk of adverse events between H2RAs/PPIs vs. placebo (33–35). Moreover, studies found that H2RAs could exert potent modulatory effects on the cells with innate and adaptive immunity to defend against the virus (36). Although the finding could support the acute URTI treatment with H2RAs, more evidence of effectiveness and safety are required, especially when using in infants. Additionally, we presumed H2RAs/PPIs might also be used as an empiric prophylactic therapy in infants as the infants tended to be seriously ill when being admitted to the hospitals. Nevertheless, the appropriateness of the clinical practices is still questioned. Further studies are urgently requested to evaluate the effectiveness and safety of these practices.

The common off-label prescriptions of H2RA/PPI prescription in Chinese infants in our study warned that regulation of H2RA/PPI use was required. It would be useful and necessary to educate and inform physicians about the misuse and potential adverse effects of H2RAs/PPIs in infants. Moreover, given the uncertainty of effectiveness and safety of H2RAs/PPIs in infants, clinicians had better try alternative therapies or interventions in the first place. These may include considering conservative means, taking anti-reflux precautions, reducing aerophagy, using hypoallergenic formulae, consideration of Eosinophilic Esophagitis, reassurance, etc. (37).

Our study, however, was subjected to several limitations. Firstly, as a retrospective observational study, our analyses were based on the hospital medication records. The database included information on patient characteristics, diagnoses and drug use. This limited our study perspectives to explore the underlying factors associated with H2RA/PPI prescribing and the explanation for H2RAs/PPIs prescribed to treat patients without digestive system diseases. Secondly, characteristic and diagnosis data of admissions of patients not using H2RAs/PPIs was unobtainable. This may limit the analysis to figure out the factors associated with H2RA/PPI use in the target population. Thirdly, the bias derived from missing data or recording errors in diagnoses could lead the proportion of admissions diagnosed without digestive system diseases overestimated. Finally, as a hospital-based study in a single center, it may not be generalized to other suppliers of the pediatric medical services. However, based on the data retrieved from one of the largest children's hospitals in China, we believed that the findings could reflect the problem of H2RA/PPI use among infants and could be representative to some extent.

In conclusion, our study showed that although not approved for infants in China, H2RA/PPI prescription was common in hospitalized infants, even those admitted with non-digestive diseases. The findings highlighted the necessity of optimizing and regulating H2RA/PPI use. Further studies, such as clinical trials, are urgently needed to evaluate the effectiveness and safety of H2RAs/PPIs in infants.

## Data Availability Statement

The data analyzed in this study is subject to the following licenses/restrictions: the datasets analyzed for this study are available from the corresponding authors on a reasonable request. Requests to access these datasets should be directed to Xiaodong Guan, guanxiaodong@pku.edu.cn.

## Ethics Statement

The studies involving human participants were reviewed and approved by Ethics Committee of Peking University Health Science Center, Beijing, China. Written informed consent from the participants' legal guardian/next of kin was not required to participate in this study in accordance with the national legislation and the institutional requirements.

## Author Contributions

XG, QL, and YC: conceptualization. YZ, LX, and CC: methodology. XG and LS: validation. YZ, HW, AY, and ZX: formal analysis. XG and QL: resources and supervision. YZ, HW, and AY: data curation. YZ: writing—original draft preparation and project administration. XG: writing—review and editing. XG and YZ: funding acquisition. All authors read and approved the final manuscript.

## Conflict of Interest

The authors declare that the research was conducted in the absence of any commercial or financial relationships that could be construed as a potential conflict of interest.
