# Supplementary material for: Acid Suppression Use Among Infants in One Tertiary Children's Hospital in China, 2015–2018: A Retrospective Observational Study
Source: Front Pediatr. 2021 May 21;9:679203. doi: 10.3389/fped.2021.679203 (PMC8175967; doi:10.3389/fped.2021.679203)
Supplement: Supplementary file 1 [file Table_1.docx]

Supplementary Material

**Supplementary Table 1. Demographic information of the study sample and H2RA/PPI use**

| **Variables** | **Inpatient n (%)** |
| --- | --- |
| **Gender** |  |
| Male | 4621 (64.6) |
| Female | 2537 (35.4) |
| **Age** |  |
| <28 days | 1028 (14.4) |
| [28days, 1 year) | 4180 (58.4) |
| [1 year,2 years] | 1950 (27.2) |
| **Medical insurance** |  |
| URBMI | 2078 (29.0) |
| NRCMS | 2090 (29.2) |
| None | 2990 (41.8) |
| **Acid suppression medications** | |
| **PPIs** | **5148 (71.9)** |
| *Omeprazole* | *4981 (69.6)* |
| *Lansoprazole* | *81 (1.1)* |
| *Esomeprazole* | *1 (0.0)* |
| *Pantoprazole* | *85 (1.2)* |
| **H2RAs** | **2010 (28.1)** |
| *Cimetidine* | *2010 (28.1)* |
| **Diagnoses with digestive system diseases (ICD-10 K00-K93)** | **3040 (42.5)** |
| *K00-K14 Diseases of oral cavity, salivary glands and jaws* | *45 (0.6)* |
| *K20-K31 Diseases of esophagus, stomach and duodenum* | *914 (12.8)* |
| *K35-K38 Diseases of appendix* | *4 (0.1)* |
| *K40-K46 Hernia* | *289 (4.0)* |
| *K50-K52 Noninfective enteritis and colitis* | *545 (7.6)* |
| *K55-K64 Other diseases of intestines* | *292 (4.1)* |
| *K65-K67 Diseases of peritoneum* | *43 (0.6)* |
| *K70-K77 Diseases of liver* | *216 (3.0)* |
| *K80-K87 Disorders of gallbladder, biliary tract and pancreas* | *32 (0.4)* |
| *K90-K93 Other diseases of the digestive system* | *660 (9.2)* |
| **Diagnoses with no digestive system diseases (ICD-10 level 1)** | **4118 (57.5)** |
| *I Certain infectious and parasitic diseases* | *470 (6.6)* |
| *II Neoplasms* | *87 (1.2)* |
| *III Diseases of the blood and blood-forming organs and certain disorders involving the immune mechanism* | *91 (1.3)* |
| *IV Endocrine, nutritional and metabolic diseases* | *25 (0.3)* |
| *IX Diseases of the circulatory system* | *58 (0.8)* |
| *V Mental and behavioral disorders* | *12 (0.2)* |
| *VI Diseases of the nervous system* | *139 (1.9)* |
| *X Diseases of the respiratory system* | *1686 (23.6)* |
| *XII Diseases of the skin and subcutaneous tissue* | *58 (0.8)* |
| *XIII Diseases of the musculoskeletal system and connective tissue* | *32 (0.4)* |
| *XIV Diseases of the genitourinary system* | *11 (0.2)* |
| *XIX Injury, poisoning and certain other consequences of external causes* | *247 (3.5)* |
| *XVI Certain conditions originating in the perinatal period* | *631 (8.8)* |
| *XVII Congenital malformations, deformations and chromosomal abnormalities* | *496 (6.9)* |
| *XVIII Symptoms, signs and abnormal clinical and laboratory findings, not elsewhere classified* | *47 (0.7)* |
| *XXI Factors influencing health status and contact with health services* | *28 (0.4)* |
| **Frequency of acid suppression use in infants** (by the number of admissions) |  |
|  | 7158 (100.0) |

URBMI, the Urban Residents’ Basic Medical Insurance; NRCMS, the New Rural Cooperative Medical Insurance; PPIs, proton pump inhibitors; H2RAs, Histamine-2 receptor antagonists; ICD-10, International Statistical Classification of Diseases, 10^th^ Revision.
